# Two Patients with Osteochondral Injury of the Weight-Bearing Portion of the Lateral Femoral Condyle Associated with Lateral Dislocation of the Patella

**DOI:** 10.1155/2014/876410

**Published:** 2014-11-18

**Authors:** Shuji Nakagawa, Yuji Arai, Hiroaki Inoue, Satoru Atsumi, Shohei Ichimaru, Kazuya Ikoma, Hiroyoshi Fujiwara, Toshikazu Kubo

**Affiliations:** Department of Orthopaedics, Graduate School of Medical Science, Kyoto Prefectural University of Medicine, 465 Kajiicho, Kawaramachi-Hirokoji, Kamigyo-ku, Kyoto 602-8566, Japan

## Abstract

Complications of patellar dislocation include osteochondral injury of the lateral femoral condyle and patella. Most cases of osteochondral injury occur in the anterior region, which is the non-weight-bearing portion of the lateral femoral condyle. We describe two patients with osteochondral injury of the weight-bearing surface of the lateral femoral condyle associated with lateral dislocation of the patella. The patients were 18- and 11-year-old females. Osteochondral injury occurred on the weight-bearing surface distal to the lateral femoral condyle. The presence of a free osteochondral fragment and osteochondral injury of the lateral femoral condyle was confirmed on MRI and reconstruction CT scan. Treatment consisted of osteochondral fragment fixation or microfracture, as well as patellar stabilization. Osteochondral injury was present in the weight-bearing portion of the lateral femoral condyle in both patients, suggesting that the injury was caused by friction between the patella and lateral femoral condyle when the patella was dislocated or reduced at about 90° flexion of the knee joint. These findings indicate that patellar dislocation may occur and osteochondral injury may extend to the weight-bearing portion of the femur even in deep flexion, when the patella is stabilized on the bones of the femoral groove.

## 1. Introduction

Many cases of patellar dislocation involve dislocation over the lateral femoral condyle. Serious complications may include osteochondral injury of the lateral femoral condyle and patella [1]. The incidence of osteochondral injury was previously reported to be low, but recent developments in MRI and arthroscopy have shown that 40–76% of cases of patellar dislocation are complicated by osteochondral injury [2, 3]. In general, lateral dislocation of the patella occurs in the anterior non-weight-bearing portion of the lateral femoral condyle and is thought to develop when the patella is dislocated laterally from the lateral femoral condyle or the dislocated patella is reduced during mild flexion of the knee joint. We encountered 2 patients with osteochondral injury on the weight-bearing surface of the lateral femoral condyle with lateral dislocation of the patella.

## 2. Patient 1

**Figure 1:**
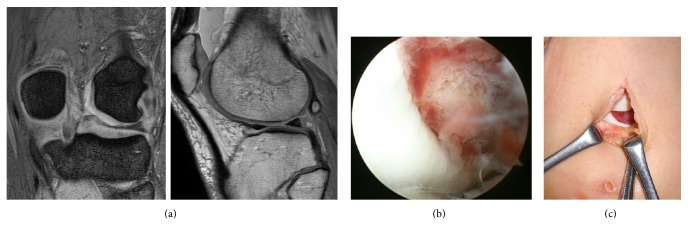
Findings in Patient 1. (a) MRI, showing an osteochondral defect in the lateral femoral condyle. (b) Arthroscopy, showing a 2 × 1.5-cm osteochondral defect in the lateral femoral condyle. (c) Arthrotomy was applied, fixing the osteochondral fragment.

An 18-year-old woman fell during tennis practice and felt pain in her left knee joint. Initial examination showed patella ballottement and positivity on the apprehension test of the patella. Three of five Carter and Wilkinson scores were positive [4]. Plain radiography of the patellofemoral joint showed Insall-Salvati ratios of 1.1 on both the left and right sides, with no evidence of patella alta. The sulcus angles on the left and right sides were 132° and 127°, respectively, showing normal morphology of the femoral groove. The congruence angles were 32° and −8°, respectively; the tilting angles (the angle between the patella width line and the tangent to the anterior condyles) [5] were 40° and 17°, respectively; and the lateral shift ratios [6] were 57% and 17%, respectively, indicating lateral subluxation and lateral inclination of the left patella. Patella tracking was along the lateral femoral condyle. Plain radiography of the entire lower extremity showed no abnormal findings. On CT scan, bone avulsion was noted in the suprapatellar pouch. Tibial tubercle-trochlear groove (TT-TG) distance was 15 mm [7]. MRI showed injury to the medial patellofemoral ligament (MPFL), as well as an osteochondral fragment in the suprapatellar pouch. T2-weighted imaging showed bone bruises in the lateral femoral condyle and the medial surface of the patellar joint. The coronal view showed a cartilage defect on the weight-bearing surface covering the distal aspect of the lateral femoral condyle (Figure 1(a)). Based on these findings, the patient was diagnosed as having left patellar dislocation and osteochondral injury of the lateral femoral condyle, and surgery was performed. On arthroscopy, cartilage injury was observed on the joint surface of the medial patella. A 2 × 1.5-cm free osteochondral fragment was present in the suprapatellar pouch, consistent with the size of the posterolateral osteochondral defect of the lateral femoral condyle (Figure 1(b)). The hematoma on the osteochondral defective region was removed, and the free osteochondral fragment was fixed with a poly-L-lactide (PLLA) pin (diameter, 2 mm; length, 20 mm) (Figure 1(c)). The patella attachment site of the vastus medialis of the quadriceps muscle was moved toward the inferolateral direction. The joint was immobilized for one week after surgery. Range of motion training and partial weight bearing were initiated at 2 weeks, and full weight bearing was initiated at 5 weeks. On the final follow-up, there was no limitation in the range of joint motion, and the patient had no knee joint pain or feeling of instability of the patella.

## 3. Patient 2

**Figure 2:**
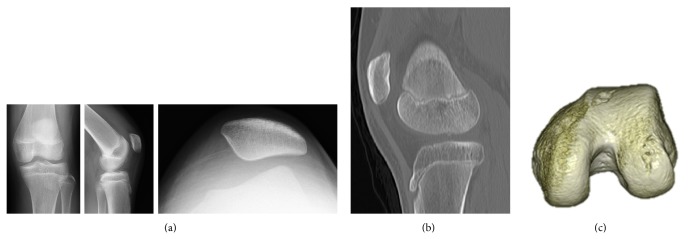
Findings in Patient 2. (a) Plain radiography, showing bone avulsion between the condyles. (b) Sagittal view of plain CT, showing an irregular 10 × 15-mm subchondral bone on the weight-bearing surface of the lateral femoral condyle. (c) 3D-CT, clearly showing the morphology of the osteochondral injury of the lateral femoral condyle.

**Figure 3:**
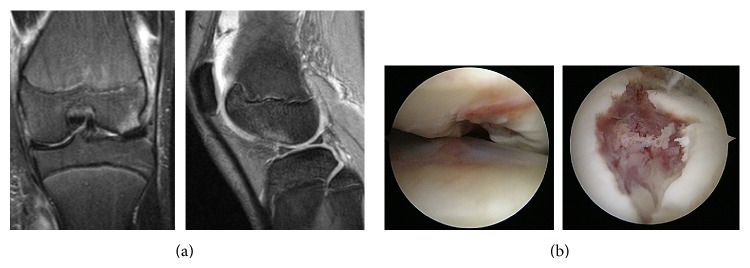
Additional findings in Patient 2. (a) T2-weighted MRI, showing a bone bruise in the lateral femoral condyle, as well as a cartilage defect of the lateral femoral condyle. (b) During extension of the knee joint, an osteochondral defect was observed in the joint surface of the weight-bearing portion of the lateral femoral condyle (left). At 90° flexion of the knee joint, a 10 × 15-mm osteochondral defect was clearly observed (right).

An 11-year-old girl fell while kicking a soccer ball and felt pain in her left knee joint. Initial examination showed patella ballottement and positivity on the apprehension test of the patella. Three Carter and Wilkinson scores were positive. Plain radiography of the knee joint showed avulsed bone between the condyles in the lateral view (Figure 2(a)). Evaluation of the patellofemoral joint showed Insall-Salvati ratios on the left and right sides of 1.3 and 1.2, respectively, indicating patella alta on left side. The sulcus angles on the left and right sides were 144° and 134°, respectively, indicating normal morphology of the femoral groove. The congruence angles were 11° and −16°, respectively; the tilting angles were 20° and 11°, respectively; and the lateral shift ratios were 21% and 9%, respectively, indicating lateral subluxation and lateral inclination of the left patella. Patella tracking was along the lateral femoral condyle. Plain radiography of the entire lower extremity showed no abnormal findings. CT scanning showed a free bone fragment in the intercondylar notch and a TT-TG distance of 18 mm. The sagittal and coronal views showed an irregular 1 × 1.5-cm subchondral bone on the weight-bearing surface of the lateral femoral condyle (Figure 2(b)). On 3D-CT, the morphology of the osteochondral injury of the lateral femoral condyle could be clearly observed (Figure 2(c)). MRI showed injury to the MPFL, and T2-weighted imaging showed bone bruises in the lateral femoral condyle and medial surface of the patellar joint. A cartilage defect in the lateral femoral condyle could also be clearly observed (Figure 3(a)). Based on these findings, the patient was diagnosed with left patellar dislocation and osteochondral injury of the lateral femoral condyle, and surgery was performed. Arthroscopy indicated that cancellous bone exposure was the site of avulsion fracture in the medial margin of the patella. A free osteochondral fragment, present in the suprapatellar pouch, was removed. A 1.5 × 1-cm osteochondral defect was present in the weight-bearing joint surface portion of the lateral femoral condyle (Figure 3(b)). Granulation was noted in the defective region and was treated with curettage and microfracture. The MPFL injury was treated with anatomical MPFL reconstruction using a semitendinosus tendon [8]. Partial weight bearing was initiated 4 weeks after surgery, and full weight bearing was initiated at 7 weeks. No limitation of the range of motion of the joint was observed on final follow-up, and the patient had no pain of the knee joint or feeling of instability of the patella.

## 4. Discussion

Osteochondral injury associated with lateral dislocation of the patella is caused in the lateral femoral condyle and patella by excess pressure of collision between the lateral femoral condyle and patella, induced by lateral traction of the patella with contraction of the quadriceps muscle in a knee valgus position. It is difficult to diagnose osteochondral injury by plain radiography alone. The incidence of osteochondral injury associated with patellar dislocation was estimated to be about 5% [9]. Although osteochondral injury was observed in 34 of 48 patients using arthroscopy, it was diagnosed in only 11 patients by plain radiography [10]. Accordingly, many reports have recommended MRI and CT to diagnose osteochondral injury [11], with osteochondral defects, not crack or fissure, observed in about 70% of these patients [12]. Plain radiography failed to clearly observe the osteochondral fragment in Patient 1 but did show it between the condyles in Patient 2. Although plain radiography could not easily determine the origin of the osteochondral fragment in either patient, MRI clearly identified both the osteochondral fragment and its origin in both. On CT, the origin was clearly observed in the coronal and sagittal reconstructed images, and irregular subchondral bone was clearly observed in the lateral femoral condyle on 3D-CT, enabling determination of the morphology of the osteochondral defect prior to surgery.

The patella starts to make contact with the trochlea at 20° flexion of the knee joint, with the contact point shifting toward the distal side of the femoral groove as flexion increases [13]. The patella fits and is stabilized on the femoral groove, resulting in greater stability in deep than in shallow flexion. Therefore, patellar dislocation generally occurs in mild flexion at a position with less bony stabilization, and patellar dislocation-associated osteochondral injury occurs in the non-weight-bearing portion anterior to the lateral femoral condyle and patella. Osteochondral injury is thought to be caused upon patellar dislocation or reduction of the dislocation. In contrast, osteochondral injury of the weight-bearing portion of the lateral femoral condyle is rare and has only been reported occasionally. One report described seven cases of osteochondral injury of the femoral weight-bearing surface caused by patellar dislocation [14], and another described a 23-year-old man with osteochondral injury of the femoral weight-bearing surface [15]. Fitting of the patellofemoral joint was favorable in Patient 1, but she exhibited general joint laxity. In Patient 2, patella alta was observed, in addition to general joint laxity, showing a stronger predisposition towards patellar dislocation than in Patient 1. Since osteochondral injury in both patients occurred in the weight-bearing portion of the lateral femoral condyle, the patella and lateral femoral condyle likely contacted at about 90° flexion when the patella was dislocated or reduced. Patellar dislocation is thought to occur in a knee valgus position, suggesting that injury to the lateral femoral condyle may be due to contusion against the lateral tibia plateau. Patellar dislocation may occur even in deep flexion, in which the patella is stabilized by bone, and osteochondral injury may extend to the weight-bearing portion of the femur.

## 5. Summary

Both patients exhibited a predisposition towards patellar dislocation, with osteochondral injury occurring in the weight-bearing portion of the lateral femoral condyle upon patellar dislocation. Patellar dislocation may occur even in deep flexion, in which the patella is stabilized by bone, and osteochondral injury may extend to the weight-bearing portion of the femur.
